# Chronic Mild Stress Modulates Activity-Dependent Transcription of BDNF in Rat Hippocampal Slices

**DOI:** 10.1155/2016/2592319

**Published:** 2015-12-31

**Authors:** Raffaella Molteni, Andrea C. Rossetti, Elisa Savino, Giorgio Racagni, Francesca Calabrese

**Affiliations:** Dipartimento di Scienze Farmacologiche e Biomolecolari, Università degli Studi di Milano, Via Balzaretti 9, 20133 Milano, Italy

## Abstract

Although activity-dependent transcription represents a crucial mechanism for long-lasting experience-dependent changes in the hippocampus, limited data exist on its contribution to pathological conditions. We aim to investigate the influence of chronic stress on the activity-dependent transcription of brain-derived neurotrophic factor (BDNF). The *ex vivo* methodology of acute stimulation of hippocampal slices obtained from rats exposed to chronic mild stress (CMS) was used to evaluate whether the adverse experience may alter activity-dependent BDNF gene expression. CMS reduces BDNF expression and that acute depolarization significantly upregulates total BDNF mRNA levels only in control animals, showing that CMS exposure may alter BDNF transcription under basal conditions and during neuronal activation. Moreover, while the basal effect of CMS on total BDNF reflects parallel modulations of all the transcripts examined, isoform-specific changes were found after depolarization. This different effect was also observed in the activation of intracellular signaling pathways related to the neurotrophin. In conclusion, our study discloses a functional alteration of BDNF transcription as a consequence of stress. Being the activity-regulated transcription a critical process in synaptic and neuronal plasticity, the different regulation of individual BDNF promoters may contribute to long-lasting changes, which are fundamental for the vulnerability of the hippocampus to stress-related diseases.

## 1. Introduction

One of the most remarkable features of the hippocampus is its ability to shape its functions and adapt to environmental changes through different mechanisms allowing neurons to adjust their properties according to their activity. These characteristics are crucial not only because of the role of this brain region in synaptic plasticity in the context of learning and memory [1], but also considering that a deficit in this skill might result in pathologic conditions. For example, given the high sensitivity to stress of the hippocampus [2–4], different studies have shown an association between hippocampal dysfunctions and stress-related diseases, such as major depression or posttraumatic stress disorders [5, 6]. Activity-regulated transcription plays a crucial role in hippocampal function [7] and may be altered under pathologic conditions [8]. A key gene for these mechanisms encodes the neurotrophin brain-derived neurotrophic factor (BDNF) that, in addition to supporting neuronal survival during CNS development [9, 10], represents an important mediator of neuronal plasticity in adulthood [11, 12]. Activity-dependent regulation of BDNF occurs through complex transcriptional mechanisms, with at least eight distinct promoters that drive the transcription of distinct mRNAs, each containing an alternative 5^I^ exon spliced to a common 3^I^ coding exon [13–15]. Although* Bdnf* promoters are differently responsive to neuronal activation [16–18], limited data are available to explain how pathological conditions may affect activity-dependent BDNF transcription. Therefore, the aim of our study was to investigate the activity-dependent transcription of the neurotrophin in the hippocampus of rats exposed to chronic stress. In depth, we used the* ex vivo* methodology of the acute stimulation of hippocampal slices obtained from rats exposed to a chronic unpredictable stress (CMS) paradigm to evaluate the different responsiveness to depolarization in terms of BDNF expression and signaling. This experimental approach replicates many aspects of the* in vivo* context as slices largely preserve the tissue architecture of the brain region they originated from and maintain neuronal activities with intact functional local synaptic circuitry. Hence, they are ideal platforms for dissection of molecular pathways underlying neuronal dysfunction.

## 2. Methods

General reagents were purchased from Sigma-Aldrich (Milan, Italy), and molecular biology reagents were obtained from Applied Biosystems Italia (Monza, Italy), Bio-Rad Laboratories S.r.l. Italia (Segrate, Italy), Santa Cruz Biotechnology (Santa Cruz, CA, USA), and Cell Signaling Technology (Danvers, MA, USA).

### 2.1. Animals

Adult male Sprague-Dawley rats (Charles River, Calco, Italy) weighing 300–350 g were used throughout the experiments. Rats were housed in groups of 3 per cage under standard conditions (12 h light/dark cycle with food and water* ad libitum*) and were exposed to daily handling for 2 weeks before any treatment. All animal handling and experimental procedures were approved by the University of Milan Institutional Animal Care and Use Committee and adhered to the Italian legislation on animal experimentation (Decreto Legislativo 116/92), the EU (*EU Directive 2010/63/EU*), and the National Institutes of Health Guide for the Care and Use of Laboratory Animals. All efforts were made to minimize animal suffering and to reduce the number of animals used.

### 2.2. Chronic Mild Stress Paradigm

**Figure 1:**
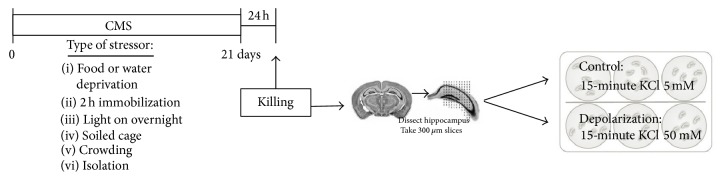
Experimental paradigm. Adult male Sprague Dawley rats were subjected to the stress procedure for 3 weeks and then sacrificed by decapitation after 24 hours from the last stressor. The brains were rapidly removed for hippocampal slices preparation. The slices, after a period of adaptation in oxygenated Krebs buffer, were incubated for 15 minutes in presence or absence of KCl 50 mM to test the effects of* ex vivo* depolarization. After the incubation period, hippocampal slices were collected, frozen on dry ice, and stored at −80°C until the molecular analyses.

For chronic mild stress, animals were randomly divided into stressed and no-stressed groups (*n* = 6 in each experimental group). Sham (no-stressed) animals were kept undisturbed in their home cages during the entire experiment except for handling manipulation every 2 days during weighing, while stressed (CMS) rats were instead exposed for 3 weeks to a variable sequence of mild, unpredictable stressors, whose application started at different times every day to minimize habits and therefore predictability. The stressors used were the following: 24 h food deprivation, isolation overnight, 2 h restraint, 24 h empty water bottle, soiled cage overnight, light on overnight, and light on and overcrowding overnight. The impact of CMS was demonstrated by a significant loss of body-weight gain, paralleled by reduced food and water consumption, as well as by reduced preference for sucrose solution with respect to control animals [19]. Twenty-four hours after having been subjected to the last stressor, animals were sacrificed by decapitation and the brains rapidly removed for the hippocampal slices preparation (Figure 1).

### 2.3. Preparation of Hippocampal Slices

Hippocampal slices were prepared (Figure 1) as described by Gardoni and colleagues [20]. Briefly, removed brains were immediately placed into chilled (4°C) oxygenated Krebs buffer and after removing the meninges, hippocampi were rapidly dissected and quickly sliced with a McIlwain tissue chopper. The slices (300 *μ*m) were then placed for 30 minutes in custom-made chambers continuously equilibrated with O_2_ 95%–CO_2_ 5% (v/v) oxygenated Krebs buffer. To induce activity-dependent transcription, potassium chloride depolarization was used. After the equilibration period, slices prepared by CTRL and STRESS rats were incubated for 15 min in presence or absence of KCl 50 mM before being collected, frozen on dry ice, and stored at −80°C until the molecular analyses. The *n* for the two different experimental conditions was 6 for acute depolarization treatment and 3 for the physiological situation (Krebs buffer).

### 2.4. RNA Preparation and Quantification of BDNF mRNA Expression by Real-Time RT Quantitative PCR

In order to measure BDNF mRNA levels, total RNA was isolated from hippocampal slices by single step guanidinium isothiocyanate/phenol extraction using PureZol RNA isolation reagent (Bio-Rad Laboratories S.r.l. Italia) according to the manufacturer's instructions and quantified by spectrophotometric analysis. The samples were then processed for real-time polymerase chain reaction (PCR) to assess BDNF mRNA levels as previously reported [21]. Briefly, a 2 *μ*g aliquot of each sample was treated with DNase to avoid DNA contamination and subsequently reverse transcribed using a High-Capacity cDNA Archive commercial kit (Applied Biosystems Italia, Monza, MI, Italy). The real-time PCR reaction was performed using the ABI Prism 7000 Sequence Detection System (Applied Biosystems Italia, Monza, MI, Italy) with the TaqMan Gene Expression Master Mix (Applied Biosystems Italia, Monza, MI, Italy) and the following TaqMan Gene Expression Assay purchased from Applied Biosystems:

Total* Bdnf*: ID Rn02531967_s1;* Bdnf* transcript IV: ID Rn01484927_m1;* Bdnf* transcript VI: ID Rn01484928_m1;* Bdnf* transcript IXa forward primer: TGGTGTCCCCAAGAAAGTAA and reverse primer: CACGTGCTCAAAAGTGTCAG.

After an initial step at 50°C for 2 min and at 95°C for 10 min, 40 cycles of PCR were performed. Each PCR cycle consisted of heating the samples at 95°C for 15 s to enable the melting process and then for 1 min at 60°C for the annealing and extension reaction. Each sample was assayed in duplicate using two independent retrotranscription products. A comparative cycle threshold (Ct) method was used to determine the relative target gene expression. Data have been expressed as percentage calculated from the expression of the target genes normalized on rat glyceraldehyde 3-phosphate dehydrogenase (GAPDH) gene expression as control gene (ID GAPDH TaqMan probe: Rn99999916_s1).

### 2.5. Preparation of Protein Extracts

Hippocampal slices were homogenized in a glass-glass potter in cold 0.32 M sucrose buffer pH 7.4 containing 1 mM HEPES, 0.1 mM EGTA, and 0.1 mM PMSF, in presence of commercial cocktails of protease (cod. 11697498001, Roche, Monza, Italy) and phosphatase (cod. P5726, Sigma-Aldrich) inhibitors. The total homogenate (H) was clarified at 1000 g for 10 min obtaining a pellet (P1) corresponding to the nuclear fraction, which was resuspended in a buffer (20 mM HEPES, 0.1 mM DTT, and 0.1 mM EGTA) supplemented with protease and phosphatase inhibitors. Total protein content was measured according to the Bradford Protein Assay procedure (Bio-Rad, Milan, Italy), using bovine serum albumin as calibration standard.

### 2.6. Western Blot Analysis

By Western blot analysis, protein extracts were used to assess the phosphorylated and the total levels of several components of BDNF-related signaling pathways in the homogenate (ERK1/2, AKT, and GSK-3*β*) and of the transcription factor CREB in the nuclear fraction.

The same amounts of total protein for all the samples (10 *μ*g for ERK1/2, AKT, and GSK-3*β*; 20 *μ*g for CREB) were run on an SDS-8% polyacrylamide gel under reducing conditions and then electrophoretically transferred onto nitrocellulose membranes (Bio-Rad, Milan, Italy). The blots were blocked with 10% nonfat dry milk and then incubated with the primary antibodies, following the manufacturer's instructions, as summarized in Table 1. Membranes were then incubated for 1 h at room temperature with the appropriate secondary antibody (see Table 1); immunocomplexes were visualized by chemiluminescence, using the ECL Western blotting kit (Amersham Life Sciences, Milan, Italy), according to the manufacturer's instructions.

Results were standardized to *β*-actin as control protein, which was detected by evaluating the band density at 43 kDa after probing the membranes with a polyclonal antibody (Sigma, dilution 1 : 10000) followed by a 1 : 10000 dilution of peroxidase-conjugated anti-mouse IgG (Sigma). Quantification of the immunoblots was performed using Quantity One software (Bio-Rad).

### 2.7. Statistical Analyses

Behavioral data were analyzed with Student's* t*-test (weight gain, panel A) and with the one-way analysis of variance (ANOVA) (weight and cage food consumption, panels B and C). Molecular data were analyzed with two-way ANOVA, with stress (No Stress versus Stress) and depolarization (KCl 5 mM versus KCl 50 mM) as independent factors and mRNA or protein levels as dependent variables. When needed, further differences were analyzed by Single Contrast post hoc test (SCPHT). Significance was assumed for *P* < 0.05. For graphic clarity, data are presented as means percent ± standard error (SEM) of control group, namely, hippocampal slices obtained from no-stressed rats and incubated with KCl 5 mM (the same concentration in Krebs buffer).

## 3. Results

### 3.1. Effects of CMS on Body Weight

**Figure 2:**
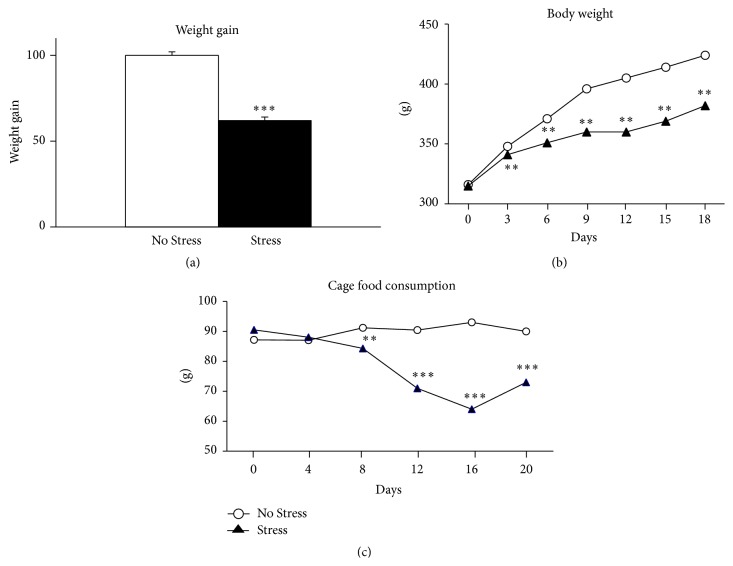
Effects of CMS on weight gain and food consuming behavior. Animals subjected to the stress procedure showed a decrease in body weight, presented as a direct comparison with the control group at 21 days (a) and as a time course during the stress period (b). (c) Showing the profile of food consumption during the 21 days of CMS procedure. ^*∗∗*^ *P* < 0.01, ^*∗∗∗*^ *P* < 0.001 versus No Stress animals. Student's* t*-test and one-way ANOVA.

We first established the effectiveness of the adverse manipulation by measuring body weight. As shown in Figure 2(a), animals exposed to 3 weeks of CMS showed significantly less weight gain when compared with control animals (*P* < 0.001, Student's* t*-test) starting from the third day of stress (Figure 2(b), one-way ANOVA), an effect that may be due also to the reduction of food consumption (Figure 2(c), one-way ANOVA).

Moreover, we previously showed that the exposure to 3 weeks of CMS induced a significantly reduced preference for sucrose solution [19]. These changes are clear indicators of the efficacy of the stressful manipulation.

### 3.2. Analysis of* Bdnf* Gene Expression

**Figure 3:**
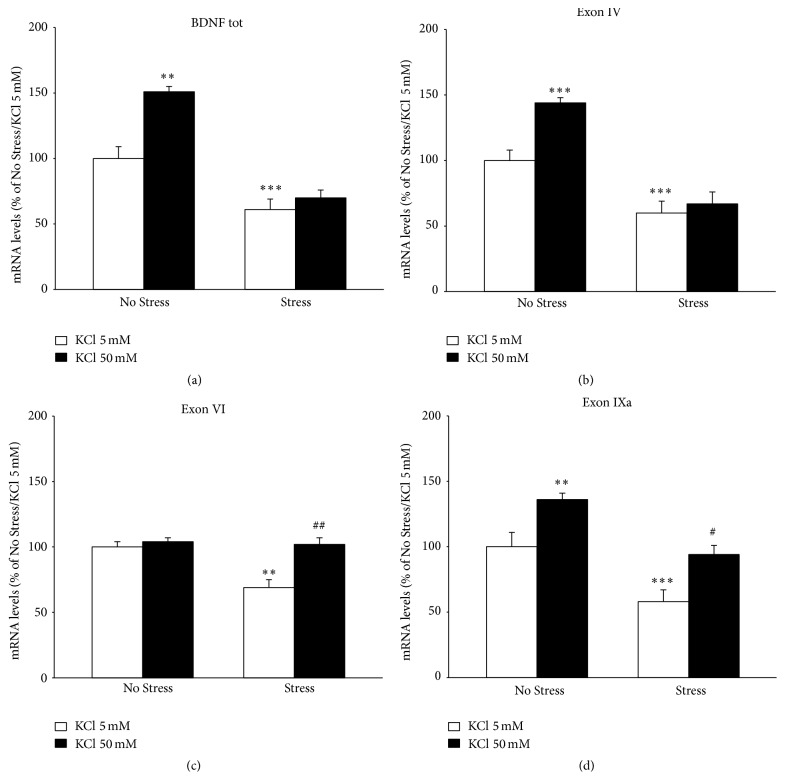
Analyses of BDNF gene expression. The mRNA levels of the total form of BDNF (a), BDNF isoform IV (b), isoform VI (c), and isoform IXa were measured by qRT-PCR in hippocampal slices obtained from unstressed (No Stress) or chronically stressed (Stress) rats exposed to KCl-induced depolarization (KCl 50 mM). The data, shown as a percentage referring to control group (No Stress/KCl 5 nM), are the mean ± SEM of independent determinations. ^*∗∗*^ *P* < 0.01, ^*∗∗∗*^ *P* < 0.001 versus No Stress animals/KCl 5 nM; ^#^ *P* < 0.05, ^##^ *P* < 0.01 versus Stress/KCl 5 nM. Two-way ANOVA with SCPHT.

As a first step, we evaluated the effect of chronic stress on total* Bdnf* (exon IX) gene expression and the CMS paradigm was found to significantly modulate the neurotrophin (*F*_1,18_ = 32.240, *P* < 0.001; ANOVA). In deep, as shown in Figure 3(a), total* Bdnf* mRNA levels were reduced in hippocampal slices prepared from stressed rats (−39% versus No Stress/KCl 5 mM, *P* < 0.001; SCPHT). When hippocampal slices were exposed to depolarizing concentration of KCl, total* Bdnf* mRNAs were significantly modulated (*F*_1,18_ = 7.888, *P* < 0.01). Indeed, BDNF expression increased in slices obtained from unstressed rats (+51% versus No Stress/KCl 5 mM, *P* = 0.01; SCPHT), whereas no changes were found in stressed rats (+9% versus Stress/KCl 5 mM, *P* > 0.05; SCPHT). In order to gain further insight into the different responsiveness to KCl, the expression profile of some neurotrophin transcripts, namely, exons IV, VI, and IXa, was investigated. Similar to what was observed for total* Bdnf*, chronic stress significantly reduced the expression of all these isoforms (Figure 3(b), isoform IV: −40%, *F*_1,15_ = 80.819, *P* < 0.001; Figure 3(c), isoform VI: −31%, *F*_1,15_ = 12.719, *P* < 0.01; Figure 3(d), isoform XIa: −42%, *F*_1,15_ = 29.455, *P* < 0.001). Their gene expression was also affected by depolarization (isoform IV: *F*_1,15_ = 15.548, *P* < 0.01; isoform VI: *F*_1,15_ = 16.542, *P* < 0.01; isoform XIa: *F*_1,15_ = 21.278, *P* < 0.001), but with different effect. Incubation with 50 mM KCl significantly increased isoform IV mRNA levels in control rats (Figure 3(b) +44% versus No Stress/KCl 5 mM, *P* < 0.001; SCPHT) but not in stressed animals (+7% versus Stress/KCl 5 mM). Conversely, under depolarizing conditions the expression of exon VI was upregulated in stressed rats (Figure 3(c) +33% versus Stress/KCl 5 mM, *P* < 0.01; SCPHT) but not in the control group (+4% versus No Stress/KCl 5 mM). Lastly, isoform IXa mRNA levels were increased after depolarization in both unstressed (+36% versus Stress/KCl 5 mM, *P* < 0.01) and stressed animals (+36% versus Stress/KCl 5 mM, *P* < 0.05), as shown in Figure 3(d).

### 3.3. Analysis of BDNF Mediated Signaling

**Figure 4:**
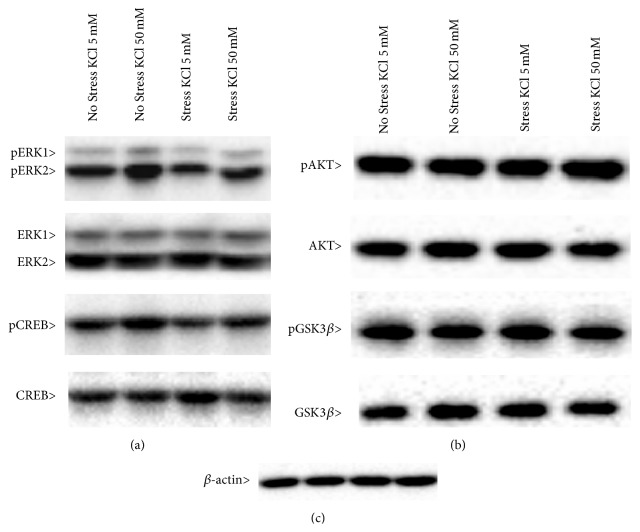
Representative Western blot analyses of the phosphorylated forms of ERK1, ERK2, and CREB (a) and of AKT and GSK3*β* (b) and their total forms (a and b). *β*-actin was used as internal standard (c). Experimental conditions are described in Methods.

**Figure 5:**
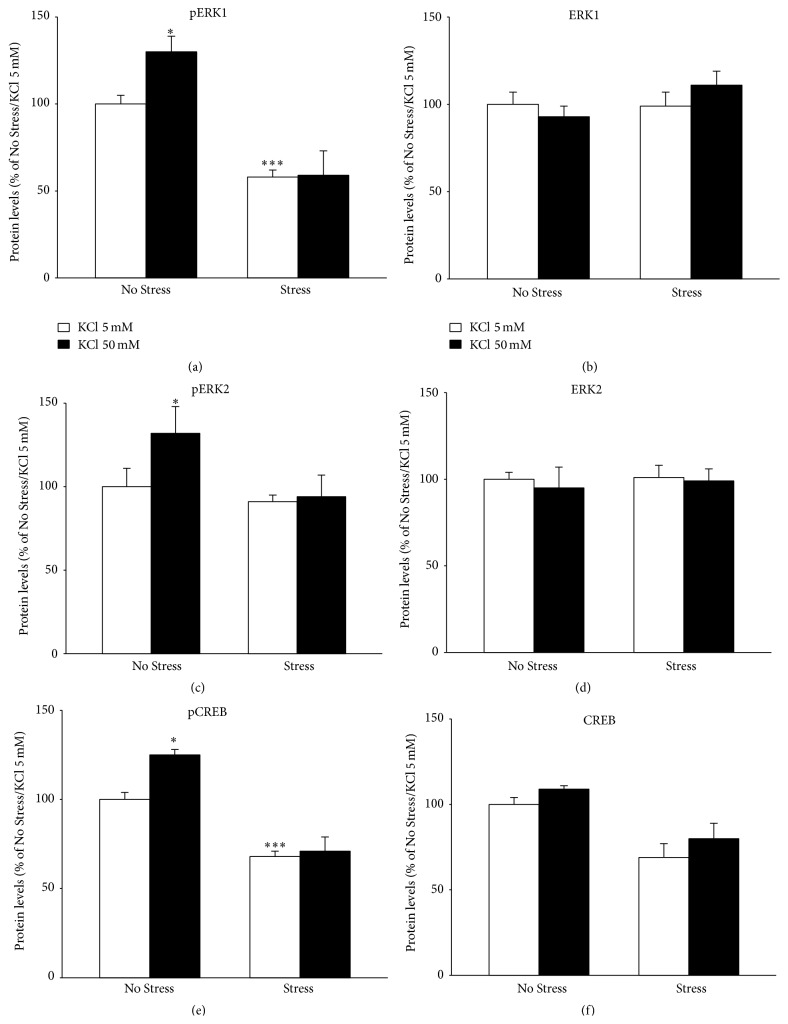
Protein analyses of BDNF mediated signaling: ERK1/2 kinases and CREB. The protein levels of the phosphorylated forms of ERK1 (a), ERK2 (c), and CREB (e) and their total forms (b, d, and f, resp.) were measured by Western blot analyses on protein extracts obtained from hippocampal slices obtained from unstressed (No Stress) or chronically stressed (Stress) rats exposed to KCl-induced depolarization (KCl 50 mM). The data, shown as a percentage referring to control group (No Stress/KCl 5 nM), are the mean ± SEM of independent determinations. ^*∗*^ *P* < 0.05, ^*∗∗∗*^ *P* < 0.001 versus No Stress animals/KCl 5 nM. Two-way ANOVA with SCPHT.

Afterwards, we examined whether the different activity-dependent transcription of* Bdnf* seen in CMS rats was paralleled by changes in signaling pathways related to the neurotrophin. We analyzed the expression and the activation (phosphorylated form) of ERK1/ERK2 (Tyr^204^/Tyr^187^), Creb (Ser^133^), and AKT (Ser^473^) and its downstream target GSK-3*β* (Ser^9^) in protein extracts obtained from hippocampal slices, under basal conditions or following KCl-induced depolarization (Figure 4). Although total levels of these signaling proteins were not modulated by CMS or by acute depolarization, we found that the phosphorylation of ERK1 and ERK2 (Figures 5(a) and 5(c)) was significantly affected by stress (pERK1: *F*_1,11_ = 41.084, *P* < 0.001; pERK2: *F*_1,12_ = 7.457, *P* < 0.05; ANOVA) and depolarization (pERK1: *F*_1,11_ = 6.432, *P* < 0.05; pERK2: *F*_1,11_ = 1.041, *P* < 0.05; ANOVA). In depth, pERK1 levels (Figure 5(a)) were significantly reduced in hippocampal slices obtained from stressed animals (pERK1 −42% versus No Stress/KCl 5 mM, *P* < 0.001). Moreover, KCl-induced depolarization increased the phosphorylated forms of both proteins in hippocampal slices obtained from unstressed rats (Figures 5(a) and 5(c)) (pERK1 +30% versus No Stress/KCl 5 mM, *P* < 0.05; pERK2 +32% versus No Stress/KCl 5 mM, *P* < 0.05; SCPHT), but not in slices obtained from animals exposed to CMS.

Moreover, we investigated the expression levels and the activation (Ser^133^ phosphorylation) (Figures 5(e) and 5(f)) of the transcription factor CREB, which is a crucial downstream element in BDNF-related signaling and a positive regulator of neurotrophin transcription [7]. We observed that both CMS and the acute depolarization displayed significant main effects on pCREB (*F*_1,10_ = 58.179, *P* < 0.001 and *F*_1,10_ = 58.179, *P* < 0.05, resp.). Similar to what was observed for total BDNF and isoform IV expression, pCREB levels were reduced in slices obtained from stressed rats (−32% versus No Stress/KCl 5 mM, *P* < 0.001, SCPHT), whereas they were increased in response to depolarization only in control animals (+25% versus No Stress/KCl 5 mM, *P* < 0.05; Figure 5(e)). Any effect on the levels of the total form of CREB (Figure 5(f)) was found.

**Figure 6:**
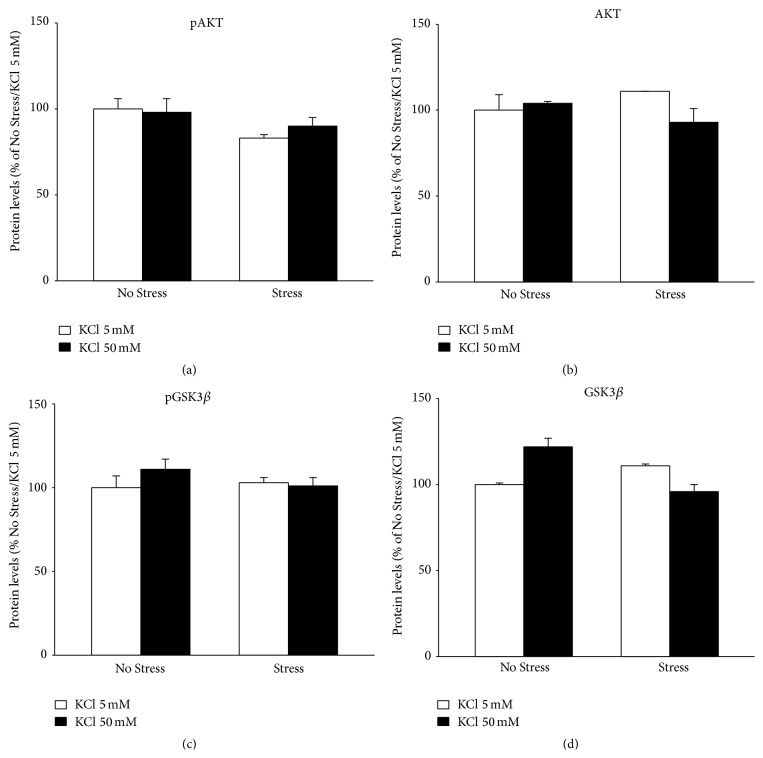
Protein analyses of BDNF mediated signaling: AKT and GSK3*β*. The protein levels of the phosphorylated forms of AKT (a) and GSK3*β* (c) and their total forms (b and d, resp.) were measured by Western blot analyses on protein extracts obtained from hippocampal slices obtained from unstressed (No Stress) or chronically stressed (Stress) rats exposed to KCl-induced depolarization (KCl 50 mM). The data, shown as a percentage referring to control group (No Stress/KCl 5 nM), are the mean ± SEM of independent determinations. Two-way ANOVA.

Conversely, neither chronic stress nor the acute depolarization was able to modulate the phosphorylation and the total levels of AKT or GSK-3*β* in the hippocampal slices (Figure 6).

## 4. Discussion

The results of our experiments disclose a novel and functional level of regulation of BDNF transcription by chronic stress. Indeed our data demonstrate not only that CMS paradigm affects basal BDNF expression but also that it has functional consequences on its activity-dependent regulation.

Different studies have examined the regulation of* Bdnf* under chronic stress, a condition that may reproduce key features of depression [22]. The interpretation of these data is not univocal, since, sometimes, opposite results have emerged based on differences in the experimental paradigm, including timing, length, and type of stressors used [23, 24]. For example, it has been showed [24] that 3 weeks of stress induced a significant increase of the protein levels of BDNF in hippocampus. Even if this effect might seem to be in contrast with our results, several reasons could explain this discrepancy, such as the different stability of the mRNA compared to protein. Another possibility could be that the stress exposure may induce an increase of the translation rate leading to a decrease of mRNA and a concomitant upregulation of the protein levels. Anyway, as explained below, also in the paper of Naert and colleagues [24] the prolonged stress altered the response to a subsequent acute challenge [24] that induced a decrease of BDNF protein levels (while, according to our data, an increase was observed in the control rats).

Our results are in line with the “classic” view, according to which CMS may lead to functional impairment through a decreased expression of neurotrophic molecules, such as BDNF [25].

However, our study provides evidence for a novel degree of regulation, demonstrating that activity-dependent modulation of the neurotrophin is impaired in the hippocampus of CMS rats. Since activity-dependent transcription represents a plastic mechanism for sustaining specific neurotrophin functions such as cognition, learning, and memory [26], the impairment of such mechanism in CMS rats may contribute to reduced plasticity and diminished ability to cope with under challenging conditions. Such defect is primarily sustained by changes in the modulation of exon IV, the major activity-dependent transcript in the hippocampus [15] whose deficits have been associated with a depressive phenotype [27, 28]. Indeed, while basal effect of CMS on total* Bdnf* reflects parallel modulation of all the isoforms examined, their analysis, following neuronal activation, provides further insight into mechanisms that may be affected by CMS. Only the expression profile of isoform IV completely reflects the modulation of total* Bdnf*. The increased mRNA levels of this transcript after depolarization are in line with the well-characterized Ca^++^-dependent modulation of its promoter [16, 29, 30] and might represent a “positive” response to enhance specific functions. In line with this hypothesis, results obtained in our laboratory have shown that activity-dependent transcription of the neurotrophin is facilitated by chronic treatment with antidepressants [21], and isoform IV specifically participates in the restorative properties of antidepressant in a genetic model of anxiety and depression [31]. Among the calcium-responsive elements mapped in* Bdnf* promoter IV, the cAMP/Ca^++^-response element (CaRE3/CRE) appears particularly important for the depolarization-induced transcription [17, 32, 33]. Our data on pCREB support this mechanism and clearly show that the activity of the transcription factor may be compromised by chronic stress.

Chronic stress also leads to a significant impairment of the MAPK pathway activation that represents a crucial point of convergence between different extracellular signals. This effect may result from reduced activity-dependent release of BDNF as well as from depolarization-induced changes of neurotransmitters release. Notably, the activation profile of ERK1/ERK2 in our paradigm paralleled the modulation of total* Bdnf* and of isoform IV, thus suggesting that the changes of these kinases may contribute to the alterations found in activity-dependent* Bdnf* transcription. Conversely, any effects on the activation and on the total levels of GSK and AKT were observed. These results seem to be in contrast with other studies [34–36] showing that the stress exposure influences the function of these pathways, but direct comparison between those results and ours is not recommended because of the different experimental conditions used.

Differently to isoform IV, isoform VI is modulated in an opposite manner, with its transcription being upregulated in CMS hippocampal slices exposed to depolarization. This suggests that the systems responsible for isoform VI activity-dependent transcription become more active even though CMS* per se* reduces its mRNA levels. Glucocorticoid hormones, which have an inhibitory control on exon VI transcription [37, 38], may eventually contribute to CMS-induced reduction. Conversely, since different intracellular systems participate in activity-dependent transcription of exon VI [39], it can be inferred that the enhanced levels of its mRNA levels in stimulated slices from CMS rats might result from the contribution and cooperation of multiple pathways differently modulated by stress and depolarization. A different influence of CMS on* Bdnf* activity-dependent transcription was observed for isoform IXa, whose mRNA levels were upregulated by depolarization in both unstressed and stress rats. Given the current lack of information on the regulation of this transcript, we cannot speculate on the mechanisms sustaining the observed effect but only highlight that BDNF transcripts may undergo different stress activity-dependent changes, which may hold implications for the diverse functions that are controlled by the neurotrophin.

To sum up, by using the* ex vivo* methodology of acute stimulation of hippocampal slices, we demonstrated that the activity-dependent modulation of BDNF expression is significantly affected by CMS exposure, thus disclosing a novel functional level of regulation of the neurotrophin by chronic stress. Given the importance of neuronal activity-regulated transcription as a critical process in synaptic and neuronal plasticity, the ability of adverse events to differently modify its control on individual BDNF promoters might be a finely regulated and flexible mechanism that contributes to long-lasting, experience-dependent changes in the hippocampus. Alternatively, the different regulation of BDNF promoters in our paradigm could result in altered translation, trafficking, and activation of signal transduction pathways that may eventually underline divergent consequences for hippocampal structure and function. Further investigations of these mechanisms may provide useful information on upstream or downstream molecular processes that, by contributing to stress-related disorders, may be a potential target for pharmacological intervention.

## Figures and Tables

**Table 1:** Antibodies conditions used in the Western blot analyses.

Gene	Primary antibody	Secondary antibody
Phospho-ERK1/2 Y204/Y187 (42–44 kDa)	1 : 1000 (Cell Signaling; **#4370**) 4°C, O/N	Anti-mouse, 1 : 2000, RT, 1 h
ERK1/2 (42–44 kDa)	1 : 5000 (Sigma; **M3807**), RT, 2 h	Anti-rabbit, 1 : 5000, RT, 1 h
Phospho-CREB S133 (43 kDa)	1 : 1000 (Cell Signaling; #**4276**), 4°C, O/N	Anti-rabbit, 1 : 2000, RT, 1 h
CREB (43 kDa)	1 : 1000 (Cell Signaling; #**9197**), 4°C, O/N	Anti-rabbit, 1 : 2000, RT, 1 h
Phospho-AKT S473 (60 kDa)	1 : 1000 (Cell Signaling; #**4060**), 4°C, O/N	Anti-rabbit, 1 : 2000, RT, 1 h
AKT (60 kDa)	1 : 1000 (Cell Signaling; #**9272**), RT, 2 h	Anti-rabbit, 1 : 1000, RT, 1 h
Phospho-GSK3*β* S9 (46 kDa)	1 : 1000 (Cell Signaling; #**9336**), 4°C, O/N	Anti-rabbit, 1 : 5000, RT, 1 h
GSK3*β* (46 kDa)	1 : 2500 (BD Transduction; **610201**) RT, 2 h	Anti-mouse, 1 : 5000, RT, 1 h
*β*-ACTIN	1 : 10000 (Sigma; **A5441**), RT, 1 h	Anti-mouse, 1 : 10000, RT, 1 h

## Abbreviations

BDNF: Brain-derived neurotrophic factor
CREB: cAMP-response element binding protein
CMS: Chronic mild stress
ERK1/2: Extracellular signal-regulated kinases 1/2
GAPDH: Glyceraldehyde 3-phosphate dehydrogenase
GSK-3*β*: Glycogen synthase kinase 3*β*.

## References

[1] Dickerson B. C., Eichenbaum H. (2010). The episodic memory system: neurocircuitry and disorders. *Neuropsychopharmacology*.

[2] McEwen B. S. (2001). Plasticity of the hippocampus: adaptation to chronic stress and allostatic load. *Annals of the New York Academy of Sciences*.

[3] De Kloet E. R., Joëls M., Holsboer F. (2005). Stress and the brain: from adaptation to disease. *Nature Reviews Neuroscience*.

[4] McEwen B. S. (2007). Physiology and neurobiology of stress and adaptation: central role of the brain. *Physiological Reviews*.

[5] MacQueen G., Frodl T. (2011). The hippocampus in major depression: evidence for the convergence of the bench and bedside in psychiatric research. *Molecular Psychiatry*.

[6] Wingenfeld K., Wolf O. T. (2014). Stress, memory, and the hippocampus. *Frontiers of Neurology and Neuroscience*.

[7] West A. E., Chen W. G., Dalva M. B. (2001). Calcium regulation of neuronal gene expression. *Proceedings of the National Academy of Sciences of the United States of America*.

[8] Malykhin N. V., Coupland N. J. (2015). Hippocampal neuroplasticity in major depressive disorder. *Neuroscience*.

[9] Huang E. J., Reichardt L. F. (2001). Neurotrophins: roles in neuronal development and function. *Annual Review of Neuroscience*.

[10] Soulé J., Messaoudi E., Bramham C. R. (2006). Brain-derived neurotrophic factor and control of synaptic consolidation in the adult brain. *Biochemical Society Transactions*.

[11] Lu B., Pang P. T., Woo N. H. (2005). The yin and yang of neurotrophin action. *Nature Reviews Neuroscience*.

[12] Monteggia L. M. (2007). Elucidating the role of brain-derived neurotrophic factor in the brain. *American Journal of Psychiatry*.

[13] Aid T., Kazantseva A., Piirsoo M., Palm K., Timmusk T. (2007). Mouse and rat BDNF gene structure and expression revisited. *Journal of Neuroscience Research*.

[14] Pruunsild P., Kazantseval A., Aid T., Palm K., Timmusk T. (2007). Dissecting the human BDNF locus: bidirectional transcription, complex splicing, and multiple promoters. *Genomics*.

[15] Pruunsild P., Sepp M., Orav E., Koppel I., Timmusk T. (2011). Identification of cis-elements and transcription factors regulating neuronal activity-dependent transcription of human BDNF gene. *Journal of Neuroscience*.

[16] Tabuchi A., Nakaoka R., Amano K. (2000). Differential activation of brain-derived neurotrophic factor gene promoters I and III by Ca^2+^ signals evoked via L-type voltage-dependent and *N*-methyl-D-aspartate receptor Ca^2+^ channels. *The Journal of Biological Chemistry*.

[17] Tao X., Finkbeiner S., Arnold D. B., Shaywitz A. J., Greenberg M. E. (1998). Ca^2+^ influx regulates BDNF transcription by a CREB family transcription factor-dependent mechanism. *Neuron*.

[18] Park H., Poo M.-M. (2013). Neurotrophin regulation of neural circuit development and function. *Nature Reviews Neuroscience*.

[19] Guidotti G., Calabrese F., Anacker C., Racagni G., Pariante C. M., Riva M. A. (2013). Glucocorticoid receptor and fkbp5 expression is altered following exposure to chronic stress: modulation by antidepressant treatment. *Neuropsychopharmacology*.

[20] Gardoni F., Schrama L. H., Kamal A., Gispen W. H., Cattabeni F., Di Luca M. (2001). Hippocampal synaptic plasticity involves competition between Ca^2+^/calmodulin-dependent protein kinase II and postsynaptic density 95 for binding to the NR2A subunit of the NMDA receptor. *The Journal of Neuroscience*.

[21] Molteni R., Calabrese F., Cattaneo A. (2009). Acute stress responsiveness of the neurotrophin bdnf in the rat hippocampus is modulated by chronic treatment with the antidepressant duloxetine. *Neuropsychopharmacology*.

[22] Duman R. S., Monteggia L. M. (2006). A neurotrophic model for stress-related mood disorders. *Biological Psychiatry*.

[23] Calabrese F., Molteni R., Racagni G., Riva M. A. (2009). Neuronal plasticity: a link between stress and mood disorders. *Psychoneuroendocrinology*.

[24] Naert G., Ixart G., Maurice T., Tapia-Arancibia L., Givalois L. (2011). Brain-derived neurotrophic factor and hypothalamic-pituitary-adrenal axis adaptation processes in a depressive-like state induced by chronic restraint stress. *Molecular and Cellular Neuroscience*.

[25] Calabrese F., Molteni R., Riva M. A. (2011). Antistress properties of antidepressant drugs and their clinical implications. *Pharmacology and Therapeutics*.

[26] Flavell S. W., Greenberg M. E. (2008). Signaling mechanisms linking neuronal activity to gene expression and plasticity of the nervous system. *Annual Review of Neuroscience*.

[27] Molteni R., Cattaneo A., Calabrese F. (2010). Reduced function of the serotonin transporter is associated with decreased expression of BDNF in rodents as well as in humans. *Neurobiology of Disease*.

[28] Sakata K., Jin L., Jha S. (2010). Lack of promoter IV-driven BDNF transcription results in depression-like behavior. *Genes, Brain and Behavior*.

[29] Tao X., West A. E., Chen W. G., Corfas G., Greenberg M. E. (2002). A calcium-responsive transcription factor, CaRF, that regulates neuronal activity-dependent expression of BDNF. *Neuron*.

[30] Hong E. J., McCord A. E., Greenberg M. E. (2008). A biological function for the neuronal activity-dependent component of Bdnf transcription in the development of cortical inhibition. *Neuron*.

[31] Calabrese F., Molteni R., Cattaneo A. (2010). Long-term duloxetine treatment normalizes altered brain-derived neurotrophic factor expression in serotonin transporter knockout rats through the modulation of specific neurotrophin isoforms. *Molecular Pharmacology*.

[32] Shieh P. B., Hu S.-C., Bobb K., Timmusk T., Ghosh A. (1998). Identification of a signaling pathway involved in calcium regulation of BDNF expression. *Neuron*.

[33] Hang C.-H., Chen G., Shi J.-X., Zhang X., Li J.-S. (2006). Cortical expression of nuclear factor *κ*B after human brain contusion. *Brain Research*.

[34] Zhang K., Song X., Xu Y. (2013). Continuous GSK-3beta overexpression in the hippocampal dentate gyrus induces prodepressant-like effects and increases sensitivity to chronic mild stress in mice. *Journal of Affective Disorders*.

[35] Qi H., Mailliet F., Spedding M. (2009). Antidepressants reverse the attenuation of the neurotrophic MEK/MAPK cascade in frontal cortex by elevated platform stress; reversal of effects on LTP is associated with GluA1 phosphorylation. *Neuropharmacology*.

[36] Fang Z. H., Lee C. H., Seo M. K. (2013). Effect of treadmill exercise on the BDNF-mediated pathway in the hippocampus of stressed rats. *Neuroscience Research*.

[37] Schaaf M. J. M., De Kloet E. R., Vreugdenhil E. (2000). Corticosterone effects on BDNF expression in the hippocampus. Implications for memory formation. *Stress*.

[38] Hansson A. C., Sommer W. H., Metsis M., Strömberg I., Agnati L. F., Fuxe K. (2006). Corticosterone actions on the hippocampal brain-derived neurotrophic factor expression are mediated by exon IV promoter. *Journal of Neuroendocrinology*.

[39] Takeuchi Y., Miyamoto E., Fukunaga K. (2002). Analysis on the promoter region of exon IV brain-derived neurotrophic factor in NG108-15 cells. *Journal of Neurochemistry*.

